# Diseases of the Circulation

**Published:** 1906-03-17

**Authors:** 


### DISEASES OF THE CIRCULATION.

**Angina Pectoris.**—W. Russell [1] brings forward a new theory of this affection which he thinks meets many difficulties, attributing the attacks to an exaggeration of the normal abdominal reflex, which sets up peripheral vaso-motor spasm. This contraction occurs normally during digestion, but abnormal sensitiveness may be set up by habitual excess in proteid food or alcohol. When this abnormal condition exists a spasm may be set up by walking uphill, by emotion, or other slight disturbances. Russell gives an interesting critical history of the theories which have been put forward, and then goes on to explain the dilatation of the splanchnic vessels during digestion and the normal contraction of the systemic vessels which counterbalances it, as well as the abnormal and excessive contraction which occurs in overfed persons and in vessels subject to arterio-sclerosis. This sudden contraction causes an embarrassment of the heart either by the sudden strain on a feeble heart-muscle or on one deprived of blood by the spasm. Thus, whether there be cardiac degeneration or no, a reflex contraction set up by abdominal causes may, if of excessive strength, produce cardiac embarrassment and some form or other of what is called angina pectoris. In treatment it is essential to cut off the excess in those foods which lead to the abnormal sensitiveness of the reflex. Minervini [2] points out that the position which sufferers from angina usually take, if possible, is one of retroversion with the head and trunk hyper-extended, and he thinks that this position is an important thing to notice in making a diagnosis from asthmatic or uræmic attacks. Clifford Allbutt [3] lays down that true angina is not a disease of the heart, but of the first part of the aorta, and that the pain is not a pain of the cardiac area, but is found chiefly in the area of the upper sternum. The mortality depends on the health or disease of the heart. He mentions three cases of recovery where the probable lesion was aortitis, and where, as the morbid condition of the first part of the aorta passed away, the angina also vanished. He adds that dyspnœa is not a feature of angina, though respiration is more or less inhibited during an attack. Musser [4] pleads for the more common use of opium, both as a cardiac tonic, and in small doses to prevent attacks of angina by reducing the susceptibility to peripheral stimuli, and also to relieve the dyspnœa of myocarditis. This view is, to some extent, supported by the well-known value of small doses of morphia in painful forms of aortic valve lesions.

**Cardiac Degeneration.**—The Oxford discussion [5] showed how great are our difficulties in diagnosis and treatment of myocardial affections. Dreschfeld first dwelt on the heart-failure of diphtheria which may come on early or late, with many or few symptoms, and may pass away, or end either fatally or in chronic myocarditis. A serious type occurs, too, in pneumonia, in which the heart failure is due not only to the toxin, but also to the mechanical embarrassment. In these acute forms either caffeine or strychnine are more often of use than digitalis. Among types of chronic myocarditis that due to syphilis may be instanced. It is rarely produced by congenital syphilis, and is either diffused or gummatous. There may be no symptoms, or only those of heart failure. Sometimes dilatation, either alone or with hypertrophy, or an aneurysm of the wall may result, or atheroma of the pulmonary vessels may complicate it, leading to hypertrophy of the right ventricle. Yet it is rarely that we can distinguish the syphilitic from other forms, except when it improves under specific treatment. Myocarditis due to external poisons, such as alcohol, may produce dilatation or hypertrophy with dyspnœa and œdema, or symptoms of Bright's disease, whereas tobacco rarely causes dilatation or degeneration, though its temporary effects are so marked. Myocardial changes, again, may be due to Graves' and Bright's disease, gout, cancer, and diabetes, or to arterio-sclerosis. The latter condition may be the forerunner of bradycardia, tachycardia, or angina, besides definite cardiac changes. In chronic lung affections we find first hypertrophy of the right side and finally dilatation, and in emphysema the left side is also affected. Heart strain from sudden exertion may cause dyspnœa and pain, but no physical signs, or there may be a small irregular pulse or dilatation. Prolonged, excessive toil may cause no disturbance of the heart, and cases where failure does occur are often due to other causes, such as alcohol or rheumatism. Indeed, heart strain after sudden exertion, as A. Foxwell remarks, is frequently due to some previous degeneration. There may be recovery or a heart permanently capable of only slight exertion. The fatty heart includes both the condition due to fatty degeneration, which gives rise to no definite symptoms, and also that produced by fatty infiltration. In the latter case, if the muscle is healthy and large enough for its work, the infiltration with fat does no harm. In other cases the symptoms may vary from slight dyspnœal palpitation to anginal attacks, Cheyne-Stokes breathing, and sudden dilatation. Björksten [6] has examined the degenerative changes produced by bacterial toxins in rabbits. His experiments tend to show that the cardiac changes produced by infectious diseases are not so extensive as we have been led to suppose. He found isolated patches of degeneration often limited to single fibres, but their occurrence was irregular and uncertain. The longer the animal lived the more marked were the interstitial and the less marked were the degenerative changes.

[1] Brit. Med. Jour., Feb. 10. [2] Brit. Med. Jour. Epit., Feb. 3. [3] Brit. Med. Jour., Jan. 6. [4] Amer. Jour. Med. Sc., Jan. [5] Brit. Med. Jour., Oct. 21. [6] Med. Record, Dec. 2.

(*To be continued.*)

